# Efficient Delivery of dsRNA and DNA in Cultured Silkworm Cells for Gene Function Analysis Using PAMAM Dendrimers System

**DOI:** 10.3390/insects11010012

**Published:** 2019-12-20

**Authors:** Chenchen Lu, Zhiqing Li, Li Chang, Zhaoming Dong, Pengchao Guo, Guanwang Shen, Qingyou Xia, Ping Zhao

**Affiliations:** 1 Biological Science Research Center, Southwest University, Chongqing 400715, China; luchenchenlcc@163.com (C.L.); cl17783721226@163.com (L.C.); dong-zhaoming@163.com (Z.D.); guopc@swu.edu.cn (P.G.); gwshen@swu.edu.cn (G.S.); xiaqy@swu.edu.cn (Q.X.); zhaop@swu.edu.cn (P.Z.); 2 Chongqing Key Laboratory of Sericultural Science, Chongqing Engineering and Technology Research Center for Novel Silk Materials, Southwest University, Chongqing 400715, China

**Keywords:** PAMAM dendrimers, dsRNA delivery, DNA delivery, *Bombyx mori*

## Abstract

Polyamidoamine (PAMAM) dendrimers are emerging as intriguing nanovectors for nucleic acid delivery because of their unique well-defined architecture and high binding capacity, which have been broadly applied in DNA- and RNA-based therapeutics. The low-cost and high-efficiency of PAMAM dendrimers relative to traditional liposomal transfection reagents also promote their application in gene function analysis. In this study, we first investigated the potential use of a PAMAM system in the silkworm model insect. We determined the binding property of G5-PAMAM using dsRNA and DNA in vitro, and substantially achieved the delivery of dsRNA and DNA from culture medium to both silkworm BmN and BmE cells, thus leading to efficient knockdown and expression of target genes. Under treatments with different concentrations of G5-PAMAM, we evaluated its cellular cytotoxicity on silkworm cells, and the results show that G5-PAMAM had no obvious toxicity to cells. The presence of serum in the culture medium did not affect the delivery performance of DNA and dsRNA by G5-PAMAM, revealing its convenient use for various purposes. In conclusion, our data demonstrate that the PAMAM system provides a promising strategy for delivering dsRNA and DNA in cultured silkworm cells and promote its further application in individuals.

## 1. Introduction

A variety of carriers have been developed for the delivery of nucleic acids into cells to achieve gene regulation. In general, two kinds of carriers exist: viral and non-viral vectors [1]. Viral vectors have been widely used due to their high efficiency in transferring nucleic acids to cells. The risk of inducing immune responses and mutagenic effects, however, has limited their application in vivo [2,3]. Therefore, non-viral vectors have received increased attention [4,5]. Various types of non-viral vectors have emerged, such as cationic molecular, liposomes, polymeric nanoparticles, polymeric micelles, polyamidoamine (PAMAM) dendrimers, solid-lipid nanoparticles, silica nanoparticles, and carbon nanotubes, which have demonstrated different abilities in delivering DNA, small interfering RNA (siRNA), and double-stranded RNA (dsRNA) [6,7,8,9].

Cationic molecular and liposomes are traditional transfection reagents for overexpressing or knocking down the expression of target genes to in vitro investigate the function in cells [10,11]. These reagents enable nucleic acids to undergo cellular uptake, either through shielding their negative charge, or decreasing their polarity. However, lower transfection efficiency compared to viral vectors and high toxicity to cells are their major disadvantages for application in vivo [12,13]. The development of various nanoparticles has shown their stronger binding affinity for DNA and RNA molecules, providing an effective and relatively nontoxic approach for transferring nucleic acids into cells, which confers the potential for drug delivery and gene therapy to combat various diseases [12].

In cultured insect cells, a major strategy for the delivery of nucleic acids to cells is still dependent on the traditional liposomal transfection reagents [14]. For instance, to investigate the function of a gene of interest, RNA interference (RNAi) using siRNA or dsRNA is a remarkably effective tool for suppressing specific gene expression. The transport of siRNA or dsRNA, however, is largely achieved via the transfection process in insect cells [14]. To bypass the use of transfection reagents, the expression of *Caenorhabditis elegans* systemic RNAi defective-1 (SID-1) was successfully applied to stimulate the spontaneous uptake of dsRNA in several model organisms including *Drosophila melanogaster* S2 cells, *Bombyx mori* N4 and e21 cells, and *Spodoptera frugiperda* SF9 cells [15,16,17,18]. Although these efforts have accelerated the functional research mediated by RNAi in these insects, the preference of this uptake for RNA rather than DNA molecules, such as plasmid DNA, also suffers from the same issue of transfection when researchers need to deliver plasmid DNA into cells.

PAMAM dendrimers, possessing well-defined structures within a nanoscale volume, have been extensively investigated as nanovectors for DNA delivery and were reported to be able to transfer RNA molecules, providing a potential platform for various drug deliveries either in DNA- or RNA-based therapeutics [13,19,20,21]. Even though PAMAM dendrimers have been widely used in mice and humans, this application in insect cells has been limited to the best of our knowledge [22,23].

To explore the effectivity of PAMAM dendrimers in insects, we focused on the use of PAMAM in silkworm *B*. *mori*, which is an excellent lepidopteran model organism for studying genetics and genomics [24,25]. From the data presented here, we found that G5-PAMAM, one generation of PAMAM dendrimers, is able to effectively deliver dsRNA and plasmid DNA into the two widely used silkworm BmN and BmE cells without significant cytotoxicity [26,27]. G5-PAMAM-mediated delivery of dsRNA targeting to translationally controlled tumor protein (TCTP) led to specific silencing of the *TCTP* gene. Plasmids containing fluorescent-labeled EGFP-TCTP and Red-ubiquitin (Ub) proteins were also expressed in both silkworm cell lines by the transportation of G5-PAMAM. TCTP-dsRNA and Ub-dsRNA delivered by G5-PAMAM showed significant gene silencing effects for transiently expressed EGFP-TCTP and Red-Ub, respectively. G5-PAMAM-mediated delivery of luciferase reporter plasmids was also tested, which is an expansion of its application in experiments for different purposes.

## 2. Materials and Methods

### 2.1. Cell Culture

The BmE and BmN cell lines were derived from silkworm embryos and ovaries [26,27], and cultured in Grace (Gibco, Waltham, MA, USA) and TC-100 medium (Sigma, St. Louis, MO, USA) supplemented with 10% fetal bovine serum (FBS, Hyclone, Logan, UT, USA) and penicillin-streptomycin (Thermo Fisher Scientific, Waltham, MA, USA) at 27 °C, respectively.

### 2.2. Plasmids

Expression constructs for TCTP and Ub were amplified from the cDNA library of cultured silkworm cells using the primers listed in Table S1 and further cloned into an NcoI–XhoI site of pENTR11 (Invitrogen, Carlsbad, CA, USA) vector [28]. All plasmids were verified by sequencing. The pENTR11-TCTP and pENTR11-Ub were respectively inserted into the expression vectors of pPBO_ie2GW (containing N-terminal EGFP tag) and pPBO_ie2RW (containing N-terminal Red tag) by gateway reaction to construct the expression plasmids of EGFP-TCTP and Red-Ub [29].

### 2.3. Double-Stranded RNA

Double-stranded RNA (dsRNA) for TCTP and Ub was synthesized using T7 RNA polymerase (Promega, Madison, WI, USA) in vitro according to a previous report [30]. The dsRNA for the control gene luciferase (*LUC*) was also synthesized [31].

### 2.4. Gel Analysis of dsRNA and Plasmid DNA with G5-PAMAM Complexes

G5-PAMAM belongs to the fifth generation of PAMAM dendrimers with triethanolamine (TEA) core and 128 amine surface groups, was purchased from Chenyuan Co. (Shandong, China) and diluted to an appropriate concentration in Milli-Q H_2_O, with all solutions stored at −20 °C. The dsRNA and plasmid DNA were diluted with Milli-Q H_2_O. Both solutions were mixed in various ratios at normal temperature for 5 min. dsRNA-G5 and plasmid-G5 complexes were analyzed by electrophoretic mobility-shift assays in 1.2% agarose gel in standard tris-acetate-EDTA (TAE) buffer. The bands were stained by Super GelRed dye (US Everbright Inc., Silicon Valley, CA, USA) and then detected by a Gel Doc™ XR+ Gel Documentation System (BIO-RAD, Hercules, CA, USA).

### 2.5. G5-PAMAM-Mediated Delivery Assay

For the delivery assay, 5 × 10^4^ BmE or BmN cells were seeded in 24-well plates in 500 μL fresh complete medium, and complexes of dsRNA (1000 ng)-dendrimer and plasmid DNA (1000 ng)-dendrimer were also prepared. The complex solution was added to the culture medium at different concentrations and incubated for 72 h. Cells treated by dsRNA or plasmid DNA were harvested and used for real-time (RT)-PCR or fluorescence analysis, respectively.

### 2.6. RT-PCR Assay

For evaluating the RNAi efficiency after dsRNA-dendrimer treatment, BmE and BmN cells were collected for extracting total RNA and synthesizing cDNA according to a previously reported protocol [29]. All cDNA samples were normalized using the internal control of the housekeeping gene *silkworm actin 3*. RT-PCR was performed in a total reaction volume of 25 μL by using TCTP primers (Table S1). We detected 10 μL of products on 1% agarose gel and stained with Super GelRed dye.

### 2.7. Fluorescence Assay

BmE and BmN cells expressing EGFP-TCTP and Red-Ub under different concentrations of G5-PAMAM were observed by using a fluorescent microscope (Z16, Leica, Wetzlar, Germany).

### 2.8. Cell Proliferation Assay

Cell proliferation was determined using a cell counting kit-8 (CCK-8, Beyotime, Shanghai, China). Briefly, 2000 cells were seeded in 96-well plates and cultured in a final volume of 100 μL medium in the presence of different G5-PAMAM concentrations. At 24, 48, and 72 h of cultivation, 10 μL of CCK-8 solutions was added into each well and the cells were further incubated for 3 h. The absorbance was measured at 450 nm in a 96-well microplate reader. The experiments were repeated at least three times.

### 2.9. Dual Luciferase Assay

The firefly luciferase reporters pGL3-LUC (negative plasmid) and pIE2-LUC (positive plasmid), and renilla luciferase reporter pRL-VgP78M (internal plasmid) were used according to a previous report [32]. The complexes of plasmid DNA (1000 ng)-PAMAM were prepared and added to BmE and BmN cells. After 48-h incubation, cells were collected and luciferase activity was measured using a Dual-Glo luciferase assay kit (Promega, Madison, WI, USA) and a GloMax-Multi Detection System (Promega, Madison, WI, USA). All assays were performed in triplicate and data are reported as the relative luciferase activity (ratio of firefly luciferase/renilla luciferase).

## 3. Results and Discussion

### 3.1. Complex Formation between G5-PAMAM and Various Types of Nucleic Acids

**Figure 1:**
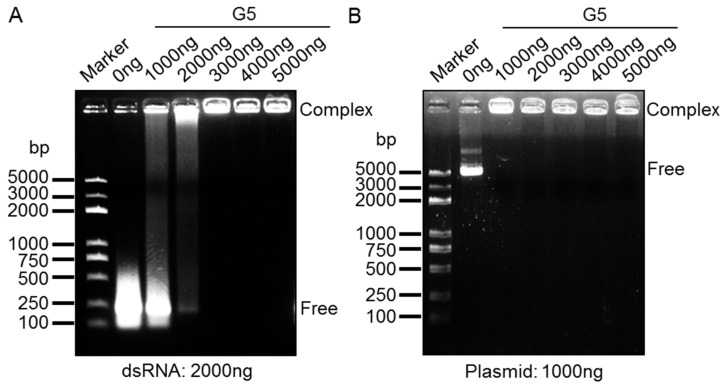
Binding assays of dsRNA and DNA with G5-polyamidoamine (PAMAM) dendrimers. (**A**) Gel retardation of G5-PAMAM dendrimers to dsRNA. Complexes were prepared from the same dsRNA and different amount of G5-PAMAM reagents, and migrated in agarose gel. (**B**) Gel retardation of G5-PAMAM dendrimers to DNA plasmid. Complexes were prepared from the same plasmid DNA and different amount of G5-PAMAM reagents, and migrated in agarose gel.

To evaluate the ability of G5-PAMAM dendrimer to deliver nucleic acids in cultured silkworm cells, we first studied the complex formation between our target molecules and G5-PAMAM in vitro using gel mobility-shift assays. Consistent with a previous report that PAMAM could form stable complexes with siRNA [23], G5-PAMAM was also able to interact with the dsRNA of *TCTP*, which led to retarded dsRNA migration in agarose gel (Figure 1A). We also observed that the complete shift of dsRNA migration was about one at a mass ratio of G5/dsRNA, which was comparable to the N/P ratio at 2.5 for PAMAM/siRNA (N/P ratio = (total terminal amines in cationic dendrimer)/(phosphates in siRNA)) [23]. We further analyzed the interaction between plasmid DNA and G5-PAMAM. We found that the plasmid DNA could be completely bound by G5-PAMAM at a mass ratio of one as well (Figure 1B). All these data reveal that G5-PAMAM dendrimer is able to effectively interact with both dsRNA and plasmid DNA at a mass ratio of one in vitro, which provides an easy method to assess the saturation degree of PAMAM dendrimers with different types of nucleic acids.

### 3.2. Efficient dsRNA Delivery in Cultured Silkworm Cells Using G5-PAMAM System

**Figure 2:**
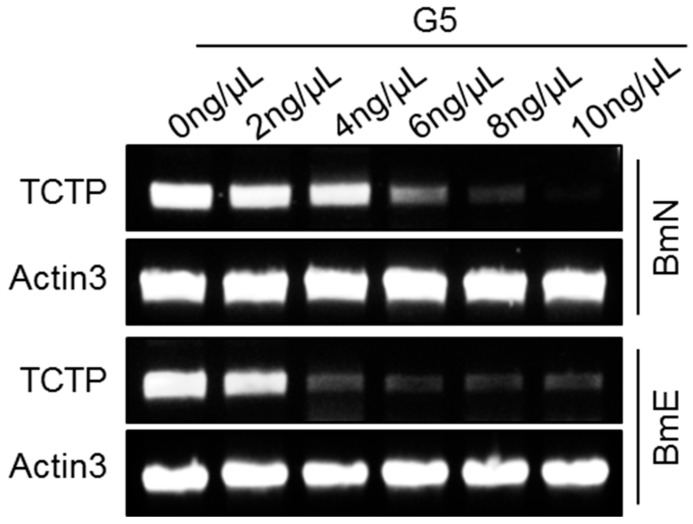
Efficient gene-specific RNAi in cultured silkworm cells using G5-PAMAM system. Semi-quantitative RT-PCR was used to evaluate RNAi efficiency of dsRNA targeting *TCTP* gene in different concentration of G5-PAMAM in cultured silkworm BmN and BmE cell lines. The expression of *BmActin3* gene was used as an internal control.

We next evaluated the gene silencing effect resulting from TCTP dsRNA delivery by G5-PAMAM in cultured silkworm cells. To estimate the efficiency of dsRNA delivery, we added the same amount of dsRNA but different G5-PAMAM into cells and produced the final amount of G5-PAMAM in a series of concentrations. After treatments with dsTCTP-G5 complexes for three days, we measured the effects of G5-mediated delivery of dsTCTP on the levels of TCTP mRNA expression. As shown in Figure 2, the mRNA levels of TCTP were detected by RT-PCR using total RNA extracted from either BmN or BmE cells. Following the increase in G5-PAMAM concentration, the knockdown efficiency significantly improved to 6 ng/μL in BmN cells and 4 ng/μL in BmE cells. We concluded that the efficient and specific down-regulation of TCTP mRNA levels in the silkworm cells is a direct consequence of the RNAi response after effective delivery of TCTP dsRNA mediated by the G5-PAMAM system.

### 3.3. Efficient DNA Delivery in Cultured Silkworm Cells Using G5-PAMAM System

**Figure 3:**
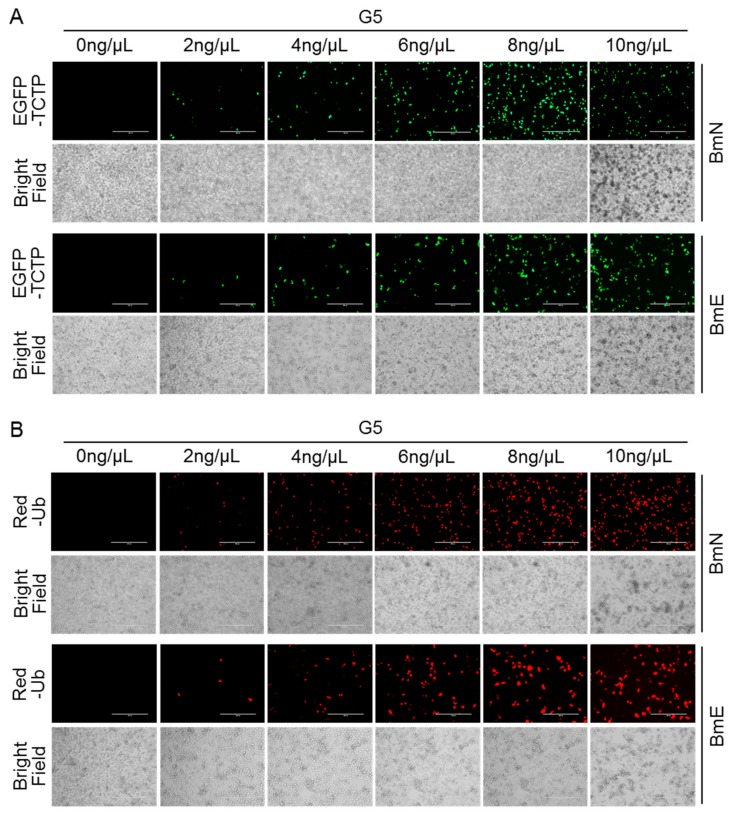
Efficient DNA delivery in cultured silkworm cells using G5-PAMAM system. (**A**) Expression profiles of EGFP-TCTP in different concentration of G5-PAMAM. The green fluorescence was observed under a fluorescent microscope. Scale bar, 400 μm. (**B**) Expression profiles of Red-Ub in different concentration of G5-PAMAM. The red fluorescence was observed under a fluorescent microscope. Scale bar, 400 μm.

Enhanced green fluorescent protein (EGFP) and red fluorescent protein (Red) expressed in prokaryotic and eukaryotic cells are capable of producing strong green and red fluorescence when excited by illumination with ultraviolet, respectively, which makes them excellent reporters for in vivo observation [33,34]. Fusion expression of fluorescent protein for a target protein has been extensively used for direct visualization of a target protein in various cells and organs using confocal microscopy. The transfection process for the fusion expression plasmids by various transfection reagents is an essential step in transporting exogenous DNA into cells. To evaluate the alternative method of DNA delivery by the G5-PAMAM system in silkworm, we labeled the silkworm *TCTP* and *Ub* genes with EGFP and Red, respectively, and cultured them with different concentrations of G5-PAMAM in both BmN and BmE cells. As shown in Figure 3, EGFP-TCTP and Red-Ub were efficiently expressed, and their expressions were largely dependent on the amount of G5-PAMAM where the optimal concentrations for DNA delivery were 6 ng/μL and 8 ng/μL. Notably, the high concentration of G5-PAMAM at 10 ng/μL would still produce some effects in cells in which agglomeration occurred when observed under a bright field microscope. These data thus indicate a novel strategy to deliver plasmid DNA using G5-PAMAM at an optimal concentration into cells in silkworm.

### 3.4. Evaluation of G5-PAMAM Dendrimers on Cellular Cytotoxicity

**Figure 4:**
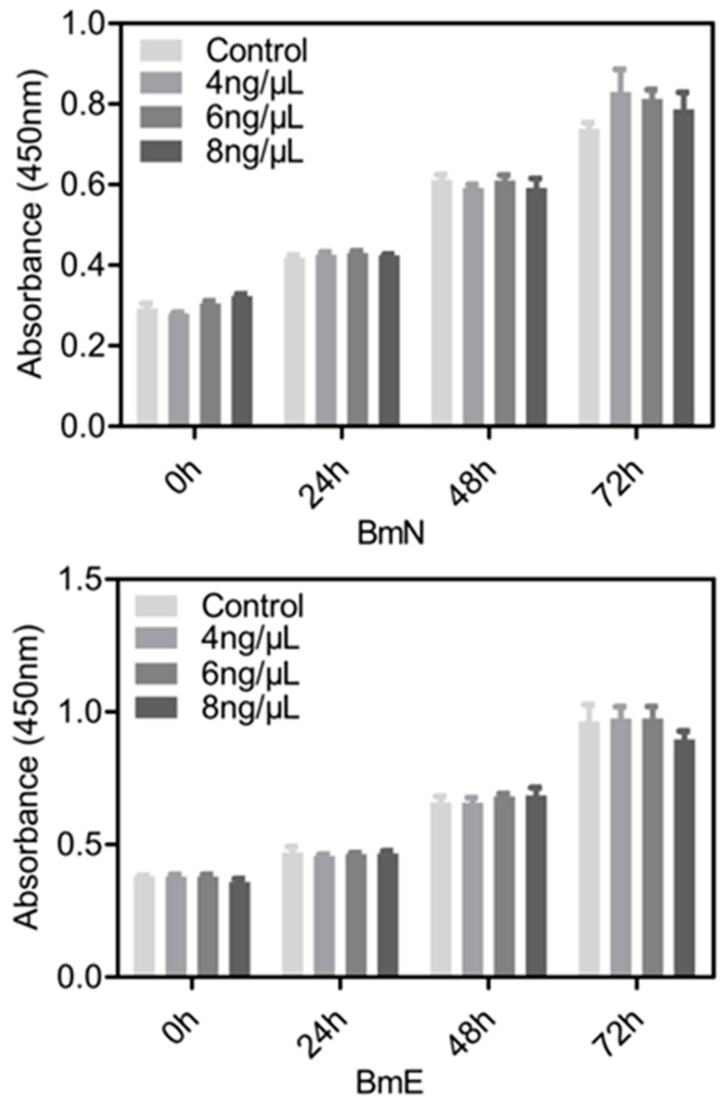
Effect of G5-PAMAM dendrimers on the cytotoxicity of cultured silkworm cells. Cell viability was determined by CCK-8 assay after incubating the cells with different concentration of G5-PAMAM in different time points. All experiments were performed in triplicate and there was no significant difference between G5 treatment and control.

We thought that the appropriate amount of G5-PAMAM would be safe for the cultured silkworm cells. To further assess the effects of G5-PAMAM on cells, we chose several concentrations of G5-PAMAM with better delivery ability but unobserved cell damages, and evaluated cell proliferation using the CCK-8 assay. When compared with the control treatment, we observed no significant effects of the G5-PAMAM concentration at 4 ng/μL, 6 ng/μL, and 8 ng/μL on cell proliferation of both BmN and BmE cells under a series of culture times (Figure 4). Therefore, the cytotoxicity of G5-PAMAM dendrimers on silkworm cells can be ignored if applying an appropriate concentration or amount. This finding agrees with results reported previously, which indicated the non-cytotoxic characteristics of PAMAM-mediated siRNA delivery in human prostate cancer (PC-3) cells [23].

### 3.5. Gene Function Assay Using G5-PAMAM System in Cultured Silkworm Cells

**Figure 5:**
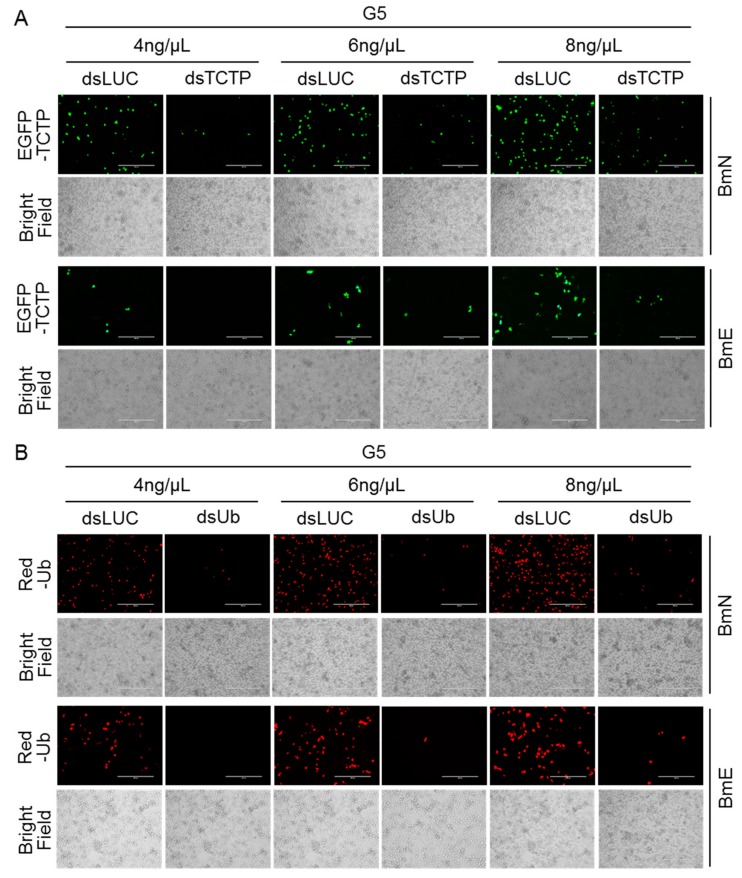
Efficient delivery of both dsRNA and DNA for gene function assay by using G5-PAMAM system. (**A**) Silencing expression of EGFP-TCTP by dsTCTP rather than dsLUC control in different concentration of G5-PAMAM. The green fluorescence was observed under a fluorescent microscope. Scale bar, 400 μm. (**B**) Silencing expression of Red-Ub by dsUb rather than dsLUC control in different concentration of G5-PAMAM. The red fluorescence was observed under a fluorescent microscope. Scale bar, 400 μm.

We confirmed the delivery capacity of the G5-PAMAM system for either dsRNA or DNA in cultured silkworm cells. We then asked whether the G5-PAMAM could simultaneously deliver both dsRNA and DNA in a gene function assay. To address this, we prepared the complexes for dsRNA targeting *TCTP* or *Ub* and plasmids of EGFP-TCTP or Red-Ub with G5-PAMAM. The complexes were then added to the medium for culturing BmN and BmE cells. When we observed the fluorescence under the microscope, we found that dsRNA targeting *TCTP* or *Ub* was able to decrease the expression of EGFP-TCTP or Red-Ub, respectively, compared with the control dsRNA treatments (Figure 5). The delivery capacity of G5-PAMAM also improved with increasing G5-PAMAM concentration. Therefore, these data indicate that G5-PAMAM system-mediated transportation of dsRNA and plasmid DNA could be a powerful tool for gene function assay in silkworm.

### 3.6. Application of G5-PAMAMSystem in Promoter Activity Analysis

**Figure 6:**
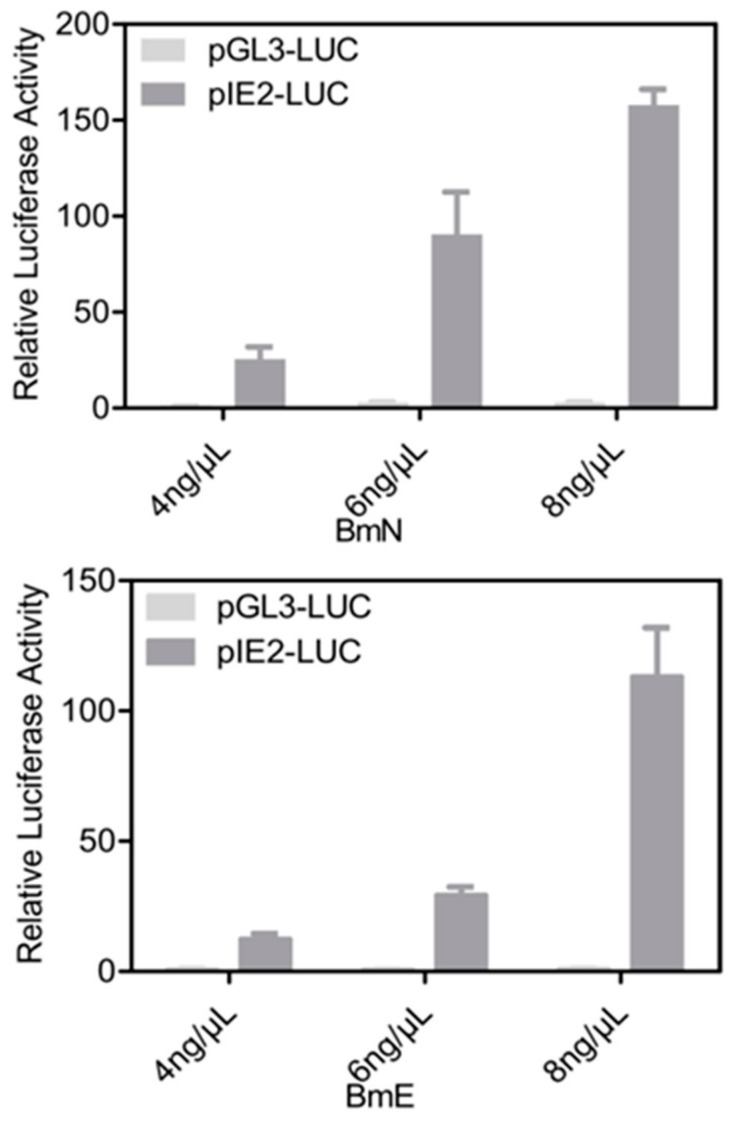
Efficient expression of luciferase by using G5-PAMAM system. The relative luciferase activities of pIE2-LUC or pGL3-LUC were measured by using a Dual-Glo Luciferase Assay Kit after normalizing with the internal pRL-VgP78M activity. All assays were performed in triplicate.

To extend the use of G5-PAMAM system to other experiments, we next detected its potential for promoter activity analysis. We used the canonical dual luciferase reporter assay in which the firefly luciferase gene is regulated either by IE2 promoter (high activity) or GL3 non-promoter (no activity) [32]. The renilla luciferase reporter pRL-VgP78M was also used as the internal control. After 48 h of culture with different G5-plasmid complexes, the dual luciferase reporter assay was performed. As shown in Figure 6, very high luciferase activities were detected for the IE2 promoter, especially at high G5-PAMAM concentration, in both BmN and BmE cells. Altogether, we concluded that the G5-PAMAM system is able to efficiently deliver plasmid DNA and dsRNA into silkworm cells. The simple operation and low toxicity to cells provides a promising and safe vector for the functional research of genes of interest in silkworm.

## 4. Conclusions

PAMAM dendrimers have been shown to act as efficient DNA and siRNA delivery systems in various mammal studies. Yet, to the best of our knowledge, almost no studies reported the use of PAMAM dendrimer-mediated dsRNA delivery in RNAi response. No reports have been published on the application of PAMAM dendrimers in insect cells. In the present work, for the first time, we used the G5-PAMAM dendrimer and explored its potential for dsRNA and plasmid DNA delivery in silkworm cells. We demonstrated that the G5-PAMAM system can efficiently deliver TCTP and Ub dsRNA as well as their expression plasmids into cells, and finally induce specific gene silencing and expression. Even in the presence of serum in the culture medium, the G5-mediated delivery of DNA and dsRNA still worked well, demonstrating its simple operation in cultured silkworm cells. The non-cytotoxicity of G5-PAMAM reagent under the optimal concentration to cells allows its safe use in various experiments. Taken together, these findings demonstrate that PAMAM dendrimers have potential as a dsRNA and DNA delivery system in silkworm and show promise for further in vivo applications.

## Supplementary Materials

The following are available online at https://www.mdpi.com/2075-4450/11/1/12/s1, Table S1. List of primers used in this study.
